# Supplementary material for: Long-Term Dynamic Changes of NMDA Receptors Following an Excitotoxic Challenge
Source: Cells. 2022 Mar 7;11(5):911. doi: 10.3390/cells11050911 (PMC8909474; doi:10.3390/cells11050911)
Supplement: Supplementary file 1 [file cells-11-00911-s001.zip › Supplementary Table S1.pdf]

Supplementary table S1

Gene	nNOS (-)	nNOS (+)	*P*
Nos1	1.07 ± 0.22	2.92 ± 0.25	0.0003
Grin1	1.15 ± 0.25	0.16 ± 0.02	0.0272
Grin2a	1.05 ± 0.16	1.02 ± 0.42	0.31
Grin2b	1.21 ± 0.42	1.11 ± 0.29	0.42
Sod2	1.03 ± 0.12	1.07 ± 0.24	0.42
Gpx1	1.06 ± 0.17	0.73 ± 0.1	0.08
Bcl2	ND	ND	
